# Oxidation Inhibits Iron-Induced Blood Coagulation

**DOI:** 10.2174/1389450111314010003

**Published:** 2013-01

**Authors:** Etheresia Pretorius, Janette Bester, Natasha Vermeulen, Boguslaw Lipinski

**Affiliations:** 1 Department of Physiology, Faculty of Health Sciences, University of Pretoria, Arcadia 0007, South Africa; 2 Joslin Diabetes Center, Harvard Medical School, Boston, MA 02215, USA

**Keywords:** Fibrinogen, blood coagulation, iron, hydroxyl radical, oxidation.

## Abstract

Blood coagulation under physiological conditions is activated by thrombin, which converts soluble plasma fibrinogen (FBG) into an insoluble clot. The structure of the enzymatically-generated clot is very characteristic being composed of thick fibrin fibers susceptible to the fibrinolytic degradation. However, in chronic degenerative diseases, such as atherosclerosis, diabetes mellitus, cancer, and neurological disorders, fibrin clots are very different forming dense matted deposits (DMD) that are not effectively removed and thus create a condition known as thrombosis. We have recently shown that trivalent iron (ferric ions) generates hydroxyl radicals, which subsequently convert FBG into abnormal fibrin clots in the form of DMDs. A characteristic feature of DMDs is their remarkable and permanent resistance to the enzymatic degradation. Therefore, in order to prevent thrombotic incidences in the degenerative diseases it is essential to inhibit the iron-induced generation of hydroxyl radicals. This can be achieved by the pretreatment with a direct free radical scavenger (e.g. salicylate), and as shown in this paper by the treatment with oxidizing agents such as hydrogen peroxide, methylene blue, and sodium selenite. Although the actual mechanism of this phenomenon is not yet known, it is possible that hydroxyl radicals are neutralized by their conversion to the molecular oxygen and water, thus inhibiting the formation of dense matted fibrin deposits in human blood.

## INTRODUCTION

It is well recognized that in the developed countries, thrombotic events are the main cause of death, mostly in the form of strokes and heart attacks. In addition, persistent presence of thrombi is observed in practically all degenerative diseases including rheumatoid arthritis, neurological disorders and cancer [1-3]. The cause of thrombosis is believed to be the activation of blood coagulation resulting in the excessive generation of thrombin, with the subsequent conversion of fibrinogen into fibrin clots. Fibrinogen (FBG), a high molecular weight (340kDa) clottable protein of human blood, plays a role in hemostasis and thrombosis, by serving as a precursor to fibrin. The structure of the fibrin network and its susceptibility to fibrinolytic degradation depend on physical, biochemical, and genetic factors that operate under various pathological conditions. Thus, the clot architecture controls the size of its pores which, in turn, determines the rate of blood perfusion making thin fibrin fibrils much less susceptible to fibrinolysis than the thick ones [4-6]. Theoretically, thrombi should be effectively removed by the intravenous infusion with plasminogen activators. However, for unexplained reasons thrombolytic therapy is effective only when installed 4-5 hours after onset of thrombosis [7]. 

Resistance of fibrin clots to enzymatic degradation, observed in numerous degenerative diseases can now be explained in terms of the effect of hydroxyl radicals (HRs), which convert soluble human FBG into insoluble and plasmin-resistant polymer. Hydroxyl free radicals are, in turn, produced by poorly chelated iron ions accumulating in the circulation from the ingested food and/or hemoglobin leaking from red blood cells (RBC). Hydroxyl radicals cause unfolding of FBG polypeptide chain(s) with the exposure of buried hydrophobic epitopes that form intermolecular bonds resistant to proteolytic degradation. Thus, it can be concluded that the commonly observed thrombolytic resistance of older thrombi might be caused by such a molecular modification induced by HRs generated under conditions of iron overload. In particular, the presence of insoluble fibrin like deposits may contribute to chronic inflammation and predisposition to thrombosis observed in the degenerative diseases. Soluble precursors, in the form of protofibrils, demonstrated in the blood of stroke patients by means of scanning electron microscopy (SEM), may also be responsible for hemorheologic disturbances observed in chronic inflammatory diseases [8, 9]. 

## MATERIALS AND METHODS

### Preparation of Fibrinogen

Human fibrinogen was purchased from Sigma Aldrich (Cat.No. F3879-250MG). This was dissolved by pouring 10 ml of warm PBS (Phosphate Buffered Saline) (pH of 7.4) over the content and gently mixed with a glass rod until completely dissolved. This stock solution can be stored at -20°C in 1-2 ml portions in plastic vials. A second stock solution was prepared with fibrinogen concentration of 2,5 mg/ml – which was also stored at -20°C. A working solution was prepared by thawing 2nd stock solution 2 at 37°C and diluting it with PBS to obtain a solution of 0.166 mg/ml. 

### Human Control Platelet Rich Plasma (PRP) and Whole Blood (WB)

Blood was obtained from 30 healthy control subjects (females ranging between 20 and 25 years) [Ethical clearance from the University of Pretoria Ethics committee was granted]. These individuals did not have any chronic conditions, did not smoke and did not use any medication. The micrographs from the healthy individuals were compared to our data base of thousands of micrographs, and found to be comparable. 

All samples for SEM were prepared using 10 µl of PRP and WB, placed directly on a glass cover slip and mixed with various compounds followed by 5 ul of human thrombin (10U/ml) (Table **1** and **2**).

### Preparation of Iron Solution to Compare Fibrinogen and PRP Smears

Previously, we showed that 5 ul of a 15mM FeCl_3_ solution caused the formation of DMDs in PRP. In the current manuscript 5 ul of a 15mM FeCl_3_ solution was therefore used in all experiments.

### Compounds Used

Hydrogen peroxide, methylene blue, sodium selenite and salicylate were prepared to various mM concentrations (Table **1** and **2**). 

### Preparation for SEM (Purified Fibrinogen, PRP and WB)

The cover slips with prepared clots were incubated at room temperature for 5 minutes and then were immersed in 0.075 M sodium phosphate buffer (pH 7.4) and finally placed on a shaker for 2 minutes. Smears were fixed in 2.5% glutaraldehyde / formaldehyde in PBS solution with a pH of 7.4 for 30 minutes, followed by rinsing 3x in phosphate buffer for five minutes before being fixed for 30 minutes with 1% osmium tetraoxide (OsO_4_). The samples were again rinsed 3x with PBS for five minutes and were dehydrated serially in 30%, 50%, 70%, 90% and three times with 100% ethanol. The material was critical point dried, mounted and coated with carbon. A Zeiss ULTRA plus FEG-SEM with InLens capabilities were used to study the surface morphology of platelets and micrographs were taken 1kV. This instrument is located in the Microscopy and Microanalysis Unit of the University of Pretoria, Pretoria, South Africa.

## RESULTS

(Table **1**) shows the mM values and µl volume for all experiments performed with the purified fibrinogen. All experiments were repeated 10 times and 10 ul portions of 0.166 mg/ml purified fibrinogen were placed on a glass cover slip and 5ul of human thrombin (10U/ml) was added to create an extensive fibrin network. (Table **2**) shows the mM values and µl volume for all experiments performed with PRP and WB. 

In control experiments Fig. (**1a** – **f**) we show purified fibrinogen, PRP and WB with additional thrombin. When thrombin is added to fibrinogen and PRP samples, an expansive fibrin network is created, where thick fibrin fibers are formed, with thin fibers sparsely distributed among these thick fibers Fig. (**1a**, **c**). In WB smears, red blood cells (RBCs) are entangled with individual thick and thin fibers Fig. (**1e**). However, when FeCl_3_ is added to PRP, fibrin fiber morphology is changed Fig. (**1b, d, f**). Ferric iron addition to fibrinogen shows dense matted deposits (DMDs) with thin fibers dispersed in between Fig. (**1b**). Ferric iron addition to PRP show a nearly solid DMD formation, where nearly no fibers are individually recognized. When ferric iron is added to WB, RBCs fused to DMDs are seen and these RBCs also tend to change form Fig. (**1f**).

We also added hydrogen peroxide and ferric iron to all three preparations. These are shown in Fig. (**2a** – **c**). In the fibrinogen preparation, thin beaded fibrin fibers are visible, but here the DMD formation is not formed. However, in the PRP sample Fig. (**2b**), no DMD formation was visible and samples appeared similar to regular control clots. Also, DMD formation was not present in WB smears Fig. (**2c**). This suggests a possible protective effect of hydrogen peroxide against ferric iron damage.

In samples treated with methylene blue Fig. (**3a** – **c**) more subtle changes were noted. In the fibrinogen experiments, thick, beaded fibrin fibers were noted Fig. (**3a**); however, in PRP samples, an amorphous clot formation was seen Fig. (**3b**) and WB samples revealed deformed RBC shapes with beaded fibrin fibers Fig. (**3c**). 

Samples treated with the anti-oxidant sodium selenite Fig. (**4a** – **c**) showed ultrastructure very similar to untreated control samples. Salicylate-treated samples are shown in Fig. (**5a** – **c**). In the purified fibrinogen experiments, dense, beaded fibrin fibers are seen Fig. (**5a**); however, PRP samples compared well to that of controls Fig. (**5b**) and RBC did not deform Fig. (**5c**); even with the addition of a lower volume of salicylate. 

## DISCUSSION

It is generally, albeit incorrectly, believed that the damaging effect of biomolecules by free radicals is caused by so-called oxidative stress. This concept stemmed from the observation that the damaged molecules are enriched with oxygen atoms. It is being neglected, however, that this can result from the reductive addition of oxygen, in the form a hydroxyl radical, across the double bond of unsaturated aliphatic and/or aromatic compounds. Another source of this widespread misunderstanding is the use of hydrogen peroxide to generated hydroxyl radicals in the presence of divalent iron ions (Fenton reaction). Iron is a transition metal that plays an essential role in many physiological functions in living organisms. Metabolism of iron in the human body is well controlled, so no large quantities of this metal are released at a given time. However, a certain proportion of iron exists in blood in a trivalent form in the so called labile iron pool [10]. In addition, ferric ions are continuously accumulating in the body from external sources, specifically from the consumption of red meats [11] and in this way may contribute to thrombosis [12].

As showed by us for the first time, ferric ions can generate the most biologically reactive hydroxyl radicals (HR) without any oxidizing agent, according to the following reaction:

$${\mathrm{Fe}}^{3+}{\mathrm{HO}}^{-}\to {\mathrm{Fe}}^{2+}{\mathrm{HO}}^{.}$$

That, in turn, causes polymerization of fibrinogen in a purified system, in plasma and/or in whole blood [13]. It is important to note that such polymerized fibrinogen fibers are very different from those produced by the enzymatic action of thrombin. It is known that the transformation of fibrin monomers into fibrin occurs by means of ionic bonds. By contrast, hydroxyl radical‑induced modification of FBG irreversibly affects its physicochemical properties rendering them more hydrophobic than the native molecules [14]. Spontaneous aggregation of soluble hydrophobic protofibrils results in the formation of dense matted deposits, which when fused with red blood cells, contributes to the resistance of thrombi to the fibrinolytic degradation [15]. 

As shown in this paper, the formation of DMDs and their subsequent fusion to RBCs can be prevented by hydrogen peroxide and certain other oxidizing agents. In the case of hydrogen peroxide this phenomenon may occur by scavenging hydroxyl radical according to the following reaction:

$${\mathrm{HO}}^{.}+H_{2}O_{2}\to O_{2}+H_{2}O+H^{+}$$

The inhibitory effect of hydrogen peroxide on the formation fibrin-like deposits, may explain the therapeutic effects of this compound reported in several papers [16-19]. As in the case of the health benefits achieved by ingestion of salicylates (aspirin), hydrogen peroxide may act by eliminating harmful hydroxyl radicals from the circulating blood. The effect of methylene blue on the non-enzymatic polymerization of fibrinogen molecules was less pronounced than that of hydrogen peroxide, possibly due to its weaker oxidation power [20]. The inhibition of fibrinogen polymerization by sodium selenite demonstrated in this paper may occur by oxidation of hydroxyl radicals and the concomitant reduction of Se^4+^ to Se^2+^. This effect is very likely to be responsible for the anticancer properties of certain selenium derivatives, particularly sodium selenite [21-25].

## CONCLUSION

The most important conclusion is that the results of this work can explain a well-known fact of failure of the so-called antioxidants in the prevention and treatment of degenerative diseases [26-28]. In fact, the most effective antioxidant, ascorbic acid is a reducing agent and thus not only alleviate oxidative stress, but often potentiate it, by contributing to the generation of hydroxyl radicals [29, 30]. Our findings clearly demonstrate the beneficial effects of oxidizing substances that prevent damage of biological macromolecules by virtue of hydroxyl radical decomposition.

## Figures and Tables

**Fig. (1):**
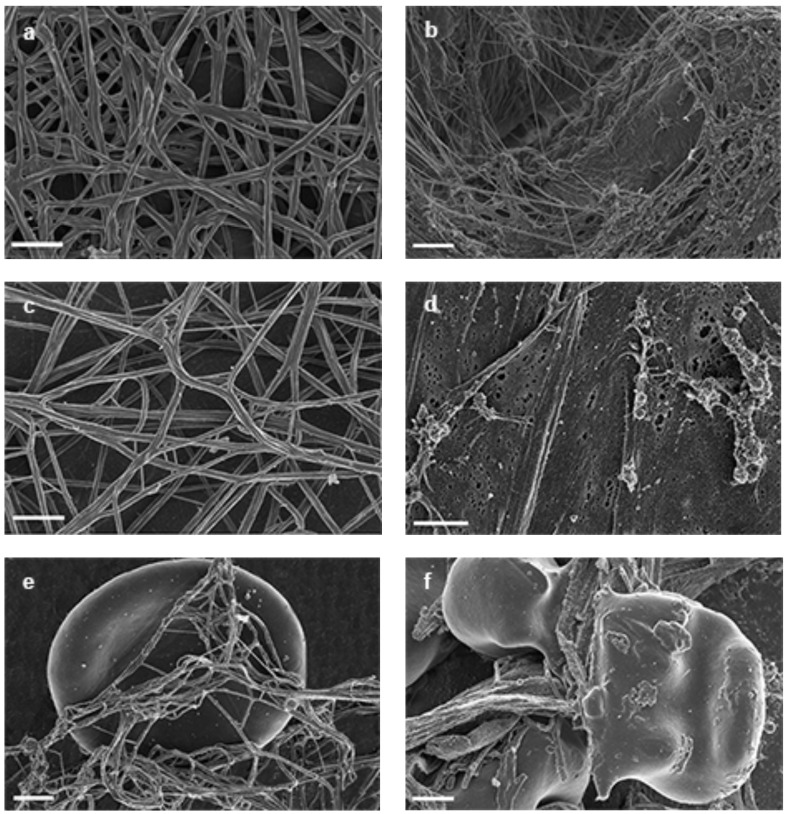
**a**) 10 µl of purified fibrinogen + 5 µl thrombin = regular clot formation (thick fibrin fibers). Scale = 1 µm. **b**) 5 µl of 15mM FeCl_3_ +10 µl of purified fibrinogen *wait 5 minutes* + 5 µl thrombin = thin fibrin fibers mixed with DMDs. Scale = 1 µm. **c**) 10 µl PRP + 5 µl thrombin = regular clot formation (thick fibrin fibers with thin fibers scattered in between). Scale = 1 µm. **d)** 5 µl of 15mM FeCl_3_ +10 µl PRP *wait 5 minutes* + 5 µl thrombin = dense matted deposits (DMD) formation. Scale = 1 µm. **e)** 10 µl WB + 5 µl thrombin = RBC surrounded by thick fibrin fibers with thin fibers scattered in between. Scale = 1 µm. **f)** 5 µl of 15mM FeCl3 + 10 µl WB *wait 5 minutes* + 5 µl thrombin = RBC fused to DMD. Scale = 1 µm.

**Fig. (2):**
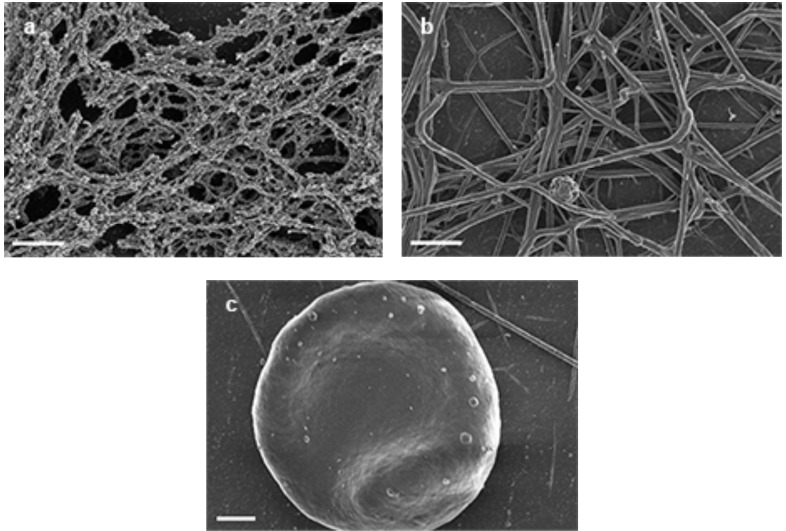
**a**) 5 µl of 15mM FeCl3 + 10 µl of 10 mM Hydrogen Peroxide *wait 5 minutes* + 10 µl purified fibrinogen + 5 µl thrombin = thin beaded fibrin fibers. Scale = 1 µm. **b**) 5 µl of 15mM FeCl_3_ + 10 µl of 10 mM Hydrogen Peroxide *wait 5 minutes* + 10 µl PRP + 5 µl thrombin = regular clot formation (thick fibrin fibers with scattered thin fibers). Scale = 1 µm. c) 5 µl of 15mM FeCl3 + 10 µl of 10 mM Hydrogen Peroxide *wait 5 minutes* + 10 µl WB + 5 µl thrombin = normal shape RBC. Scale = 1 µm.

**Fig. (3):**
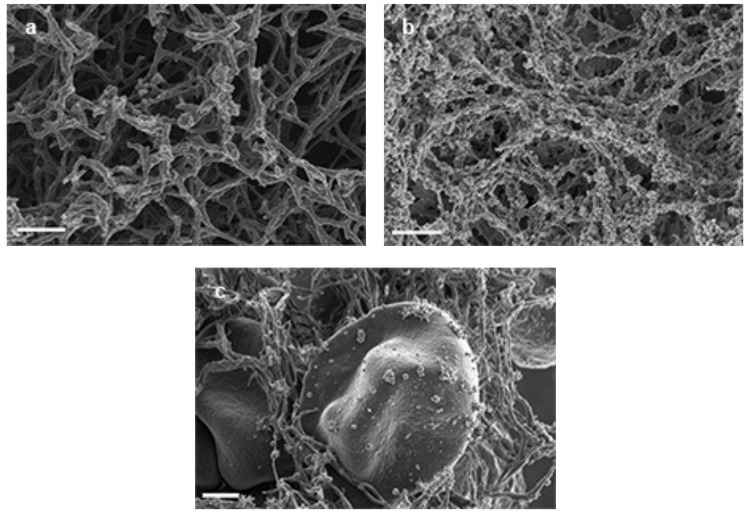
**a**) 5 µl of 15mM FeCl3 + 10 µl of 10 mM Methylene Blue *wait 5 minutes* + 10 µl of purified fibrinogen + 5 µl thrombin = thick beaded fibrin fibers. Scale = 1 µm. **b**) 5 µl of 15mM FeCl_3_ + 10 µl of 10 mM Methylene Blue *wait 5 minutes* + 10 µl PRP + 5 µl thrombin = amorphous clot formation. Scale = 1 µm. **c**) 5 µl of 15mM FeCl_3_ + 10 µl of 10 mM Methylene Blue *wait 5 minutes* + 10 µl WB + 5 µl thrombin = deformed RBC shape. Scale = 1 µm.

**Fig. (4):**
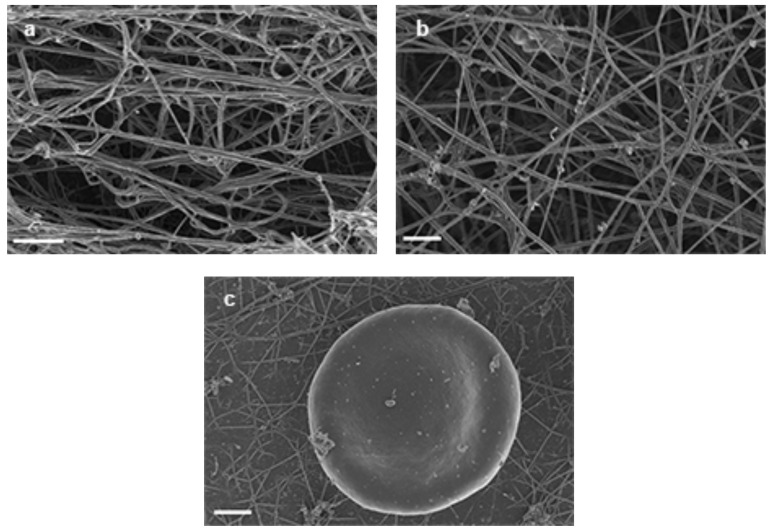
**a**) 5 µl of 15mM FeCl3 + 10 µl of 10 mM Sodium Selenite* wait 5 minutes* + 10 µl of purified fibrinogen + 5 µl thrombin = thin fibrin fibers. Scale = 1 µm. **b**) 5 µl of 15mM FeCl3 + 10 µl of 10 mM Sodium Selenite *wait 5 minutes* + 10 µl PRP + 5 µl thrombin = thin fibrin fibers. Scale = 1 µm. **c**) 5 µl of 15mM FeCl3 + 10 µl of 10 mM Sodium Selenite *wait 5 minutes* + 10 µl WB + 5 µl thrombin = normal RBC shape. Scale = 1 µm.

**Fig. (5):**
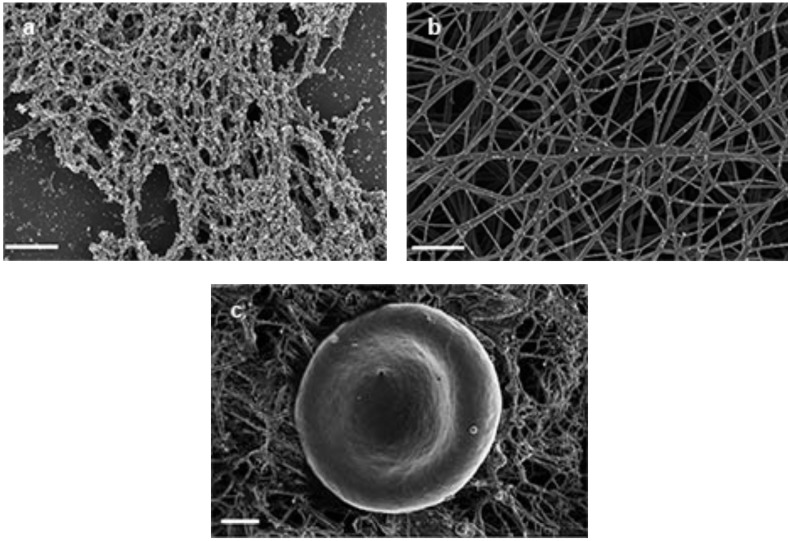
**a**) 5 µl of 15mM FeCl3 + 10 µl of 10 mM Salicylate *wait 5 minutes* + 10 µl of purified fibrinogen + 5 µl thrombin = thin fibrin fibers. Scale = 1 µm. **b**) 5 µl of a 15mM FeCl3 + 5 µl of 10 mM Salicylate wait 5 minutes + 10 µl PRP + 5 µl thrombin = dense beaded fibrin fibers. Scale = 1 µm. **c**) 5 µl of a 15mM FeCl3 + 4 µl of 10 mM Salicylate *wait 5 minutes* + 10 µl WB + 5 µl thrombin = normal RBC shape. Scale = 1 µm.

**Table 1.** Purified Human Fibrinogen Experiments

10 µl of purified human fibrinogen + 5 µl thrombin = regular clot formation (Figure 1a)
5 µl of 15mM FeCl_3_ +10 µl of purified human fibrinogen *wait 5 minutes* + 5 µl thrombin (Figure 1b)
5 µl of 15mM FeCl_3_ + 10 µl of 10 mM hydrogen peroxide *wait 5 minutes* + 10 µl of purified human fibrinogen + 5 µl thrombin (Figure 2a)
5 µl of 15mM FeCl_3_ + 10 µl of 10 mM methylene blue *wait 5 minutes* + 10 µl of purified human fibrinogen + 5 µl thrombin (Figure 3a)
5 µl of 15mM FeCl_3_ + 10 µl of 10 mM sodium selenite *wait 5 minutes* + 10 µl of purified human fibrinogen + 5 µl thrombin (Figure 4a)
5 µl of a 15mM FeCl_3_ + 10ul µl of 10 mM salicylate *wait 5 minutes* + 10 µl of purified human fibrinogen + 5 µl thrombin (Figure 5a)

**Table 2.** Platelet Rich Plasma (PRP) and Whole Blood (WB) Experiments

10 µl PRP + 5 µl thrombin (Figure 1c)
5 µl of 15mM FeCl_3_ +10 µl PRP *wait 5 minutes* + 5 µl thrombin (Figure 1d)
10 µl WB + 5 µl thrombin **= **RBC surrounded by thick fibrin fibers (Figure 1e)
5 µl of a 15mM FeCl_3_ +10 µl WB *wait 5 minutes* + 5 µl thrombin (Figure 1f)
5 µl of 15mM FeCl_3_ + 10 µl of 10 mM hydrogen peroxide *wait 5 minutes* + 10 µl PRP + 5 µl thrombin (Figure 2b)
5 µl of 15mM FeCl_3_ + 10 µl of 10 mM hydrogen peroxide *wait 5 minutes* + 10 µl WB + 5 µl thrombin (Figure 2c)
5 µl of 15mM FeCl_3_ + 10 µl of 10 mM methylene blue *wait 5 minutes* + 10 µl PRP + 5 µl thrombin (Figure 3b)
5 µl of 15mM FeCl_3_ + 10 µl of 10 mM methylene blue *wait 5 minutes* + 10 µl WB + 5 µl thrombin (Figure 3c)
5 µl of 15mM FeCl_3_ + 10 µl of 10 mM sodium selenite *wait 5 minutes* + 10 µl PRP + 5 µl (Figure 4b)
5 µl of a 15mM FeCl_3_ + 10 µl of 10 mM sodium selenite *wait 5 minutes* + 10 µl WB + 5 µl thrombin (Figure 4c)
5 µl of 15mM FeCl_3_ + 5 µl of 10 mM salicylate *wait 5 minutes* + 10 µl PRP + 5 µl thrombin (Figure 5b)
5 µl of a 15mM FeCl_3_ + 4 µl of 10 mM salicylate *wait 5 minutes* + 10 µl WB + 5 µl thrombin (Figure 5c)
